# Supplementary material for: Factors affecting calving to conception interval (days open) in dairy cows located at Dessie and Kombolcha towns, Ethiopia
Source: PLoS One. 2022 Feb 17;17(2):e0264029. doi: 10.1371/journal.pone.0264029 (PMC8853564; doi:10.1371/journal.pone.0264029)
Supplement: Attachment — Submitted filename: PONE-D-21-31364 Article 1.pdf [file pone.0264029.s003.pdf]

PLOS ONE

Survival analysis of calving to conception interval in dairy cows located at Dessie and Kombolcha towns, Ethiopia

--Manuscript Draft--

**Manuscript Number:**	PONE-D-21-31364
**Article Type:**	Research Article
**Full Title:**	Survival analysis of calving to conception interval in dairy cows located at Dessie and Kombolcha towns, Ethiopia
**Short Title:**	Survival analysis of calving to conception interval in dairy cows
**Corresponding Author:**	Alula Alemayehu Assen, MSc<br>Wollo University<br>Dessie, Amara (Amhara) ETHIOPIA
**Keywords:**	conception; days open; dairy cow; longitudinal study; postpartum; survival analysis
**Abstract:**	This study was aimed at determining the median days of calving to conception interval (days open) and identifying the major risk factors determining the days open in dairy cows. Both retrospective and longitudinal studies were conducted on dairy cows located in the Dessie and Kombolcha towns of South Wollo Zone, northeast Ethiopia from August 2019 to August 2020. The Kaplan-Meier model of survival analysis was used to determine the median days open and compare the survival distribution of each level of explanatory variables likely to influence the days open of dairy cows. Accordingly, the overall conception rate of dairy cows was 44.7 %. The median days open in the study was 154 days. There is a 16 % probability of surviving (i.e, the probability that the conception event has not yet occurred) at the end of 210 days postpartum period. The Cox proportional hazard model was used to quantify the effect of each of the explanatory variables on the days open in the first 210 days postpartum. Factors that had a significant effect ($p<0.05$) on the calving to conception interval were the season of insemination, calving season, breeding system, calving to insemination interval, and herd milk yield level. However, the peripartum, postpartum disorders, and the town of the farm are not significant ($p>0.05$). Accordingly, cows inseminated in the autumn (HR=3.91) and calved in the winter season (HR=4.93), cows less than 80 days calving to insemination interval (HR=2.50), artificially inseminated cows (HR= 1.67), and high herd milk yield level had a higher probability of becoming pregnant. In conclusion, the management practices and breeding decisions seem to be important determinants to improve the conception rate or decreasing days open in dairy cows.
**Order of Authors:**	Migbnesh Yekoye Temesgen, DVM
	Alula Alemayehu Assen, MSc
	Tarekegn Tintagu Gizaw, MSc
	Bethelehem Alemu Minalu, MSc
	Anteneh Yenehun Mersha, MSc
**Additional Information:**	
**Question**	**Response**
**Financial Disclosure**<br><br>Enter a financial disclosure statement that describes the sources of funding for the work included in this submission. Review the submission guidelines for detailed requirements. View published research articles from *PLOS ONE* for specific examples.	The author(s) received no specific funding for this work.

Powered by Editorial Manager® and ProduXion Manager® from Aries Systems Corporation

This statement is required for submission and **will appear in the published article** if the submission is accepted. Please make sure it is accurate.<br><br>**Unfunded studies**<br>Enter: *The author(s) received no specific funding for this work.*<br><br>**Funded studies**<br>Enter a statement with the following details:<br>• Initials of the authors who received each award<br>• Grant numbers awarded to each author<br>• The full name of each funder<br>• URL of each funder website<br>• Did the sponsors or funders play any role in the study design, data collection and analysis, decision to publish, or preparation of the manuscript?<br>• **NO** - Include this sentence at the end of your statement: *The funders had no role in study design, data collection and analysis, decision to publish, or preparation of the manuscript.*<br>• **YES** - Specify the role(s) played.<br><br>* typeset	
**Competing Interests**<br><br>Use the instructions below to enter a competing interest statement for this submission. On behalf of all authors, disclose any competing interests that could be perceived to bias this work—acknowledging all financial support and any other relevant financial or non-financial competing interests.<br><br>This statement is required for submission and **will appear in the published article** if the submission is accepted. Please make sure it is accurate and that any funding sources listed in your Funding Information later in the submission form are also declared in your Financial Disclosure statement.<br><br>View published research articles from *PLOS ONE* for specific examples.	The authors have declared that no competing interests exist.

Powered by Editorial Manager® and ProduXion Manager® from Aries Systems Corporation

**NO authors have competing interests**<br><br>Enter: *The authors have declared that no competing interests exist.*<br><br>**Authors with competing interests**<br><br>Enter competing interest details beginning with this statement:<br><br>*I have read the journal's policy and the authors of this manuscript have the following competing interests: [insert competing interests here]*<br><br>* typeset	
**Ethics Statement**<br><br>Enter an ethics statement for this submission. This statement is required if the study involved:<br><br>• Human participants<br>• Human specimens or tissue<br>• Vertebrate animals or cephalopods<br>• Vertebrate embryos or tissues<br>• Field research<br><br>Write "N/A" if the submission does not require an ethics statement.<br><br>General guidance is provided below. Consult the submission guidelines for detailed instructions. **Make sure that all information entered here is included in the Methods section of the manuscript.**	This study was carried out in cooperation with dairy farmers in the study areas and formalized by an official letter of cooperation obtained from the Research and Community Service Vice-President Office of Wollo University, Ethiopia. The purpose of the study was explained to all dairy farms included in the study and informed consent was sought from farm owners willing to be included in the study. The study was carried out under the existing dairy farm setting and the selection of dairy cows was for analysis cohorts. All interactions with the cows in this study were part of routine farm management where the researcher's involvement in this study was only observing and recording data in routine farm processes. Approval from an Institutional Animal Care and Use Committee (IACUC), or equivalent animal ethics committee, was not obtained as the researchers didn’t manipulate cows or the experimental design led to differential treatment from routine practices on farms.

Powered by Editorial Manager® and ProduXion Manager® from Aries Systems Corporation

**Format for specific study types**<br><br>**Human Subject Research (involving human participants and/or tissue)**<br>• Give the name of the institutional review board or ethics committee that approved the study<br>• Include the approval number and/or a statement indicating approval of this research<br>• Indicate the form of consent obtained (written/oral) or the reason that consent was not obtained (e.g. the data were analyzed anonymously)<br><br>**Animal Research (involving vertebrate animals, embryos or tissues)**<br>• Provide the name of the Institutional Animal Care and Use Committee (IACUC) or other relevant ethics board that reviewed the study protocol, and indicate whether they approved this research or granted a formal waiver of ethical approval<br>• Include an approval number if one was obtained<br>• If the study involved *non-human primates*, add *additional details* about animal welfare and steps taken to ameliorate suffering<br>• If anesthesia, euthanasia, or any kind of animal sacrifice is part of the study, include briefly which substances and/or methods were applied<br><br>**Field Research**<br><br>Include the following details if this study involves the collection of plant, animal, or other materials from a natural setting:<br>• Field permit number<br>• Name of the institution or relevant body that granted permission	
**Data Availability**<br><br>Authors are required to make all data underlying the findings described fully available, without restriction, and from the time of publication. PLOS allows rare exceptions to address legal and ethical concerns. See the PLOS Data Policy and FAQ for detailed information.	Yes - all data are fully available without restriction

Powered by Editorial Manager® and ProduXion Manager® from Aries Systems Corporation

A Data Availability Statement describing where the data can be found is required at submission. Your answers to this question constitute the Data Availability Statement and **will be published in the article**, if accepted.<br><br>**Important:** Stating 'data available on request from the author' is not sufficient. If your data are only available upon request, select 'No' for the first question and explain your exceptional situation in the text box.<br><br>Do the authors confirm that all data underlying the findings described in their manuscript are fully available without restriction?	
**Describe where the data may be found in full sentences. If you are copying our sample text, replace any instances of XXX with the appropriate details.**<br><br>• If the data are **held or will be held in a public repository**, include URLs, accession numbers or DOIs. If this information will only be available after acceptance, indicate this by ticking the box below. For example: *All XXX files are available from the XXX database (accession number(s) XXX, XXX.*).<br>• If the data are all contained **within the manuscript and/or Supporting Information files**, enter the following: *All relevant data are within the manuscript and its Supporting Information files.*<br>• If neither of these applies but you are able to provide **details of access elsewhere**, with or without limitations, please do so. For example:<br><br>*Data cannot be shared publicly because of [XXX]. Data are available from the XXX Institutional Data Access / Ethics Committee (contact via XXX) for researchers who meet the criteria for access to confidential data.*<br><br>*The data underlying the results presented in the study are available from (include the name of the third party*	All relevant data are within the manuscript and its Supporting Information files.

and contact information or URL).<br>• This text is appropriate if the data are owned by a third party and authors do not have permission to share the data.<br><br>* typeset	
Additional data availability information:	Tick here if the URLs/accession numbers/DOIs will be available only after acceptance of the manuscript for publication so that we can ensure their inclusion before publication.

Manuscript

Click here to access/download;Manuscript;calving_to_conception.doc

Survival analysis of calving to conception interval in dairy cows located at Dessie and Kombolcha towns, Ethiopia

Migbnesh Yekoye Temesgen[1¶], Alula Alemayehu Assen[1¶*], Tarekegn Tintagu Gizaw[1], Bethelehem Alemu Minalu[1], Anteneh Yenehun Mersha[2]

[1] School of Veterinary Medicine, Wollo University, Dessie, Ethiopia

[2] Pinnacle Private Limited, Addis Ababa, Ethiopia

¶These authors contributed equally to this work.

*Corresponding author

E-mail: alulaalemayehu@gmail.com (AAA)

Abstract

This study was aimed at determining the median days of calving to conception interval (days open) and identifying the major risk factors determining the days open in dairy cows. Both retrospective and longitudinal studies were conducted on dairy cows located in the Dessie and Kombolcha towns of South Wollo Zone, northeast Ethiopia from August 2019 to August 2020. The Kaplan-Meier model of survival analysis was used to determine the median days open and compare the survival distribution of each level of explanatory variables likely to influence the days open of dairy cows. Accordingly, the overall conception rate of dairy cows was 44.7 %. The median days open in the study was 154 days. There is a 16 % probability of surviving (i.e, the probability that the conception event has not yet occurred) at the end of 210 days postpartum period. The Cox proportional hazard model was used to quantify the effect of each of the explanatory variables on the days open in the first 210 days postpartum. Factors that had a significant effect ($p<0.05$) on the calving to conception interval were the season of insemination, calving season, breeding system, calving to insemination interval, and herd milk yield level. However, the peripartum, postpartum disorders, and the town of the farm are not significant ($p>0.05$). Accordingly, cows inseminated in the autumn (HR=3.91) and calved in the winter season (HR=4.93), cows less than 80 days calving to insemination interval (HR=2.50), artificially inseminated cows (HR= 1.67), and high herd milk yield level had a higher probability of becoming pregnant. In conclusion, the management practices and breeding decisions seem to be important determinants to improve the conception rate or decreasing days open in dairy cows.

Keyword: conception; days open; dairy cow; longitudinal study; postpartum; survival analysis

Introduction

Reproductive performance is the major concern of the modern dairy industry worldwide by determining the profitability of a dairy farm[1]. Poor reproductive performance is one of the most common reasons for culling in dairy herds [2]. Because, it affects the amount of milk produced per cow per day of herd life, affects the longevity of the cow in the herd, and indirectly influences herd replacement costs, breeding costs, and expenses for veterinary treatment and drugs [3]. Generally, it is believed that a calving interval of about one year is an optimal indicator of fertility and profitability of dairy herds [4]. To obtain this calving interval, a postpartum cow has to restart ovarian activity, be detected in heat, be mated, and to conceive within 85 days after calving, while producing large quantities of milk [5,6].

The calving-to-conception interval (days open), the period between parturition and the following conception of a dairy cow is the major parameter used to determine the reproductive performance and to make an economic decision in dairy herds [7]. Minimizing days open is economically beneficial by increasing milk yield relative to labor and feed costs [4,8], increase the number of calves [8] and lifetime productive days [9], as well as reduce breeding costs [8,9] and culling rates [4,8,9]. According to Louca and Legates [10] an average decrease of 2.40 kg of milk and 0.112 kg of fat for each additional day open. In addition, each added day open also extends the calving interval and decreases the number of calves available for replacement. Accordingly, the combined

milk production and decreased calves available account for an economic loss of about 0.25 to 0.71 USD cents for each day open.

Conception in a dairy cow is usually analyzed as a binary trait determined by whether or not a cow conceives after insemination following calving. Treating conception as a binary trait views pregnancy as having occurred during a defined period, hence ignoring the continuity of the conception process and the precise time of pregnancy or “failure” [11]. Analytical methods such as survival analysis is now available that offer several advantages over standard regression techniques for quantifying days open as time intervals in dairy cattle [7,12]. It accounts for continuity of the conception and does not restrict the analysis of the data to arbitrarily predefined time points. The main advantage of survival analysis is that it can retain the information from cows that are culled before conception or not pregnant by the time the data recording was completed. Thus, records from pregnant (uncensored) and non-pregnant (censored) cows can be treated jointly and included in the analysis, making proper use of all the available information. Within the field of fertility in dairy cattle, survival analysis has been applied to study the effects of diseases on days to conception [7,13] the relationship between BCS and postpartum reproductive efficiency [14], and the effect of early lactation milk yield on days open [7].

There is a scarcity of research using survival analysis has been conducted for fertility traits particularly the calving to conception interval (days open) of dairy cows in dairy farms in the tropics, particularly in Ethiopia. Information about days open and relating factors is of paramount importance to dairy farmers and as well as to extension agents, veterinarians and researchers. Moreover, it can help to develop strategies of possible interventions for improving the reproductive performance of dairy cows in general and

days open in particular. Therefore, the objective of this study was to determine the median days open and identifying the major risk factors determining the days open in dairy cows of the study area.

Materials and methods

Study area

The study was conducted in Kombolcha and Dessie towns of South wollo zone, northeast Ethiopia from August 2019 to August 2020. The towns are selected since dairy crossbreeding is widely promoted by the regional government through the distribution of pregnant crossbred heifers and the use of artificial insemination.

Study animals

The study animals are dairy cows which were found in 61 dairy farms. All study animals included in the study are cross breed cows with different age groups, parity, and body condition scores.

Sampling technique and study design

A purposive sampling technique was employed to select dairy farms and cows in the study area. Accordingly, the farms were considered based on herd size, accessibility, and availability of recording of the herd, etc. Both retrospective and prospective cohort studies were employed to collect data on days open of a cow.

Study methodology

Longitudinal study

Data: Longitudinal data were collected from dairy farms located at Dessie and Kombolcha towns, South Wollo Zone, northeast Ethiopia. The response variable of interest is the calving-to-conception interval which is called days open (DO) in the first 210 days after calving [1]. Cows that have completed the follow-up and experienced the event of interest, the conception, were considered uncensored observations. In contrast, some cows were culled, died, or were still not pregnant at the follow-up period, such animals were treated as censored [7,15]. All cows that show the event was coded one, while those with incomplete records are coded as zero (i.e., pregnant cows = 1, and non-pregnant cows = 0). Biological (herd milk yield, peri- and postpartum disorders, and calving to insemination interval) and environmental (herd, year, season of calving, and insemination) factors limiting conception were recorded for each cow [16].

Dairy cow recruitment criteria: Dairy cows that gave birth from sampled dairy farms were enrolled and followed for approximately 7 months/210 days. Cows that gave birth less than 45 days at the initial visit and whose disease history and date of the last calving known were recruited retrospectively (concurrent cohort) and allowed to join into the prospective cohort. Other cows were recruited prospectively as they give birth within the selected farms during the study period. Purchased or entrusted cows were not included in this study. The recruited cows and those cows that gave birth after the initial visit were identified and recorded at the earliest farm visit. All selected cows were regularly visited twice per month until the cows reached 7 months of postpartum. Cows are withdrawn

from the follow-up when they completed their 7 months of postpartum. When cow loss happened during the follow-up period, the date and reason for the loss were recorded [7].

Data management and analysis

The collected data was stored in the Microsoft excel database system for data management. Statistical analyses were conducted using R version 3.6.1 [17]. The median days open for cows that did conceive was determined by the Kaplan-Meier model of the survival analysis method using the {survival} package of R [18]. In addition, The association between each explanatory variable thought to influence days open was tested using the log-rank test. Each explanatory variable was categorized into 2 or more levels. The Kaplan-Meier survival curves for each level of an explanatory variable were plotted using {GGally} package, which is an extension to the {ggplot2} package of R [19].

A Cox proportional hazard model [20] was fitted to the data to quantify the effect of explanatory variables on days open in the first 210 days postpartum. Accordingly, the full model was fitted to the data including all the explanatory variables such as the season of insemination, calving season, breeding system, calving to insemination interval, and herd milk yield level, and the interactions between these variables were included in the model. Backward stepwise regression technique was used to built a multivariable cox proportional hazard model, and elimination was performed based on the Wald statistic criterion when $p>0.1$ [21]. The final model included the effects under analysis, all other significant main effects, and the interaction terms. The coefficient estimates of the survivor function and hazard ratios and 95% CI were obtained for all classes within factors included in the final model. The model is represented as follows:

$$\lambda(t,X) = \lambda_0(t)e^{\beta_1 x1+\beta_2 x2+\cdots\beta_p xp} \quad (1)$$

Where, **λ(t,x)** = hazard of an event for a cow at time t with covariate x,

λo(t) =baseline hazard function describing the hazard of an event for a hypothetical situation when all covariate values are set to zero, and

$e^{\beta_1 x1+\beta_2 x2+\cdots\beta_p xp}$ = term specific to individuals with covariate x1, x2…xp.

A p-value < 0.05 was considered statistically significant.

The set of covariates x are:

SI_i = fixed effect of season of insemination i in which the cow was last inseminated (i= 1 to 4),

CS_j = fixed effect of caving season j in which the cow has given birth (j = 1 to 4),

BS_k= fixed effect of the breeding system used for cow k (k= 1 to 2),

CIl= fixed effect of calving to insemination interval category l (l= 1 to 2),

H_MYm = fixed effect of herd milk yield category m (m= 1 to 2),

$TOWN_n$ = fixed effect of the origin (town) of the cow n (n=1 to 2),

PRE. $DIS._o$= fixed effect of prepartum disorder of the cow o (o= 1 to 2),

POST. $DIS._p$= fixed effect of postpartum disorder of the cow p (p= 1 to 2) and

The use of categorized variables was performed to look at possible nonlinear effects and to account for differences in variance within herds. Accordingly, the definition of variables included in the statistical model is presented below:

The season of insemination category (i.e., SI) was defined within every dairy cow by classifying the cow into four classes according to the season of insemination in the first 210 days after calving. Cows in class 1 were those inseminated in the autumn (September–November) season, cows in class 2, cows in class 3 and cows in class 4 were

those inseminated in the winter (December- February), spring (March-May), and summer (June–August) seasons, respectively.

The calving season (i.e., CS) was defined within every cow by classifying the cow into four classes according to the season of calving. Cows in class 1 were those calved in the autumn season, cows in class 2, cows in class 3, and cows in class 4 were those calved in the winter, summer, and spring seasons, respectively. This classification was intended to stratify the cows according to possible differences in genetic level for reproduction performance or differences in management during the rearing period.

The herd milk yield category (i.e., H_MY) was defined by classifying the herds into two classes according to the median 100-d milk yield. Herds in class H_MY_1 were those above the median, and herds in class H_MY_2 were those located below the median. This classification was intended to stratify the herds according to differences in genetic level for milk yield or differences in management during lactation. For the calculation of milk yield at 100 d of lactation, cows were required to have at least 1 test day record in the periods 5 to 50, 50 to 100, and 100 to 150 d after calving [22].

The definitions of peri- and postpartum health disorders that were used in the present study were similar to those described previously [22–24]. Calving difficulty was ranked according to the degree of assistance required (1 = no assistance, 2 = minor assistance, 3 = some force required, 4 = significant force required, and 5 = cesarean section). Cows with a calving score >2 were considered to have dystocia. The retained placenta was defined as the retention of the fetal membranes for longer than 24 hours. Septicemic metritis was defined by the presence of fever (≥39.5°C) and a watery, fetid uterine discharge during the first 10 days postpartum. Ketosis was diagnosed by the following

clinical signs within 4 weeks postpartum: anorexia, depression, and the odor of acetone on the breath. Milk fever was diagnosed by the presence of weakness and recumbency after calving. Abomasal displacement was diagnosed by the detection of a ‘ping’ sound during abdominal auscultation within 4 weeks postpartum. Clinical endometritis was diagnosed based on the presence of a visible mucopurulent vaginal discharge and/or rectal palpation of the enlarged uterus at 4 weeks postpartum.

Calving to insemination interval was grouped into two classes: short (< 80 days) and long (>= 80 days) as described by [1].

Ethics statement

This study was carried out in cooperation with dairy farmers in the study areas and formalized by an official letter of cooperation obtained from the Research and Community Service Vice-President Office of Wollo University, Ethiopia. The purpose of the study was explained to all dairy farms included in the study and informed consent was sought from farm owners willing to be included in the study. The study was carried out under the existing dairy farm setting and the selection of dairy cows was for analysis cohorts. All interactions with the cows in this study were part of routine farm management where the researcher's involvement in this study was only observing and recording data in routine farm processes. Approval from an Institutional Animal Care and Use Committee (IACUC), or equivalent animal ethics committee, was not obtained as the researchers didn’t manipulate cows or the experimental design led to differential treatment from routine practices on farms.

Results

Kaplan-Maier modeling of days open

The Kaplan-Maier estimate of the median days open in the study cow was 154 days with 95% CI (150.06, 157.94). The conception rate of dairy cows in the study was 44.7%. In addition, from the total 385 dairy cows followed in the study, 172 (44.7%) and 213 (55.3%) observations were uncensored and censored observations by 210 days postpartum, respectively (Table 1).

Table 1. Descriptive statistics of calving to conception interval (days open) under analysis

**Observations**	**Days open**
Total records, no.	385
Censored records, no	213
Minimum censoring time, day	2
Maximum censoring time, day	210
Median censoring time, day	159
Uncensored records, no.	172
Minimum failure time, day	79
Maximum failure time, day	191
Median failure time, day	154

A plot of Kaplan-Meier survival functions of the probability of postpartum conception is shown in Fig 1. The survival curves showed that there is a 16 % probability of surviving (i.e, the probability that the conception event has not yet occurred) at the end of 210 days postpartum period.

Fig 1. Kaplan-Meier estimates of survivor function of days open in dairy cows.

The results of the log-rank (Mantel-Cox) test of equality of the survival distributions for the different levels of factors on days open of dairy cows were shown in Table 2. Accordingly, factors that had a significant association ($p < 0.05$) with days open are the season of insemination, calving season, breeding system, and calving to insemination interval. However, herd milk yield, town, and pre(post)partum disorder are not significant ($p > 0.05$).

Table 2. Log-rank (Mantel-Cox) test of equality of the survival distributions for the different levels of factors on days open of dairy cows

Factor	Level	Median (SE)	95% CI	X^2	P-value
**Insemination season**	Summer	169(4.35)	(160.48, 177.52)	61.19	0.000
	Autumn	142 (4.12)	(133.93, 150.07)		
	Winter	154(4.02)	(146.11, 161.89)		
	Spring	151(4.05)	(143.06, 158.94)		
**Calving season**	Spring	171(8.57)	(154.19, 187.80)	16.02	0.001
	Winter	141(5.21)	(130.79, 151.21)		
	Summer	150(1.91)	(146.26, 153.74)		
	Autumn	157(2.47)	(152.15, 161.85)		
**Breeding system**	AI	152(2.27)	(147.54, 156.46)	5.26	0.022
	NS	159(3.45)	(152.23, 165.77)		
**Calving to Insemination**	< 80 days	144(1.29)	(141.48, 146.52)	41.87	0.000
	>=80 days	165(3.20)	(158.73, 171.27)		
**Herd milk yield (H_MY)**	H_MY1	155(2.04)	(151.00, 158.99)	1.14	0.286
	H_MY2	152(2.69)	(146.72, 157.28)		
**Town**	Dessie	152(1.94)	(148.20, 155.80)	1.27	0.260
	Kombolcha	156(2.91)	(150.06,157.94)		
**Prepartum disorder**	No	154 (2.06)	(149.96, 158.03)	0.19	0.661
	Yes	154 (12.25)	(129.99,178.00)		
**Postpartum disorder**	No	153 (1.80)	(149.47, 156.53)	0.49	0.484
	Yes	158 (2.50)	(153.10, 162.89)		

SE is the standard error of the median estimate

χ2 is the chi-square statistics

CI is the 95 % confidence interval of the estimate

NS is Natural service

AI is Artificial Insemination

Cox proportional-hazards modeling of days open

The coefficient estimates of the survivor function and hazard ratios (HR) and 95% CI of the HR were obtained for all levels of factors included in the final model fitted to the multivariable Cox proportional hazard model is presented in Table 3. Accordingly, the insemination season, calving season, breeding system, calving-to-insemination interval, and herd milk yield level have shown significant effects ($p<0.05$) on the days open of dairy cows.

Table 3. Multivariate Cox proportional hazards model of days open of dairy cows in Dessie and Kombolcha

Factors	Level	β (SE)	HR (95%CI)	P-value
**Season of insemination**	Summer		1	.000
	Autumn	1.36 (0.22)	3.91 (2.52, 6.07)	
	Winter	0.82 (0.22)	2.27 (1.48, 3.49)	
	Spring	0.47 (0.27)	1.61(0.95,2.72)	
**Calving season**	Spring		1	.000
	Autumn	0.61 (0.28)	1.84 (1.07,3.16)	
	Winter	1.59 (0.47)	4.93 (1.96, 12.41)	
	Summer	1.15 (0.28)	3.16(1.84,5.43)	
**Breeding system**	NS		1	.020
	AI	0.51 (0.19)	1.67 (1.16, 2.41)	
**Calving to insemination interval**	>=80 days		1	.000
	<80 days	0.92 (0.17)	2.50 (1.78, 3.51)	
**Heard milk yield (H_MY)**	H_MY2		1	0.002
	H_MY1	-0.62 (0.19)	0.54 (0.37,0.79)	

β is regression parameter of the survivor function

SE is the standard error of the median

HR is the Hazard ratios within variable are given relative to the hazard for the reference class, which is set to 1.0

CI is the 95 % confidence interval

NS is Natural service

AI is Artificial Insemination

The median days open for cows inseminated in summer, autumn, winter, and spring was found to be 169, 142, 154, and 151 days, respectively. A significant difference ($p<0.05$) was found among cows inseminated in different seasons (Table 2). The estimated hazard ratios show a nonlinear effect of the season of insemination on days open. Cows inseminated in autumn (HR=3.91), winter (HR=2.27), and spring (HR=1.61) had a higher probability of becoming pregnant than in summer (Table 3). The survivor function for days open was plotted for cows categorized by the season of insemination (Fig 2). The plot revealed that the survival curve for cows inseminated in autumn is consistently smaller than survival curves for cows inseminated in other seasons, respectively.

Fig 1. Kaplan-Meier estimates of survivor function for the days open of dairy cows with the season of insemination category

The calving season has a significant association ($p< 0.05$) with days open of a cow (Table 2). Dairy cows that calved in winter (HR=4.93), summer (HR=3.16), and autumn (HR=1.84) had a greater chance of becoming pregnant than those that calved in the spring, which is the dry season in the area (table 3). The survivor function for days open was plotted for cows categorized by their calving season revealed that cows inseminated in winter, summer, and autumn are consistently smaller survival curves than cows inseminated in spring (Fig 3).

Fig 2. Kaplan-Meier estimates of survivor function for the days open of dairy cows with the season of calving category

The median days open in cows with less than 80 days and greater or equals to 80 days calving to insemination interval were 144 and 165 days, respectively. This showed a 21 days extension of days open in cows that have greater or equal to 80 days calving to

insemination interval than in cows that account less than 80 days calving to insemination interval. There was a significant difference ($p<0.05$) among calving to insemination interval strata on days open (Table 2). Accordingly, cows that have less than 80 days calving to insemination interval had a higher probability (HR=2.50) to conceive than cows that have greater than or equal to 80 days calving to insemination interval (Table 3). The survival function curve of days open among the calving to insemination interval factor is presented in fig 4.

Fig 3. Kaplan-Meier estimates of survivor function for the days open with calving to insemination category

The median days open for natural service (NS) exposure was 159 days, compared to 152 days open for Artificial insemination (AI) exposed cows (Table 2). There was a 7 days extension in days open for cows with natural service relative to the artificial insemination breeding system. A significant difference ($p<0.05$) was found among the breeding system used on days open. Compared to cows exposed to natural services, cows exposed to artificial insemination were 1.67 times more likely to become pregnant or to have decreased days open (Table 3). The association between the breeding system used on days open of dairy cows is demonstrated using the survival function curve (Fig 5).

Fig 4. Kaplan-Meier estimates of survivor function for days open with the breeding system used category

There was no significant difference ($p>0.05$) between herd milk yield and days open (Table 2). However, a significant effect ($p<0.05$) of herd milk yield on days open adjusted for other covariates in the multivariable Cox proportional hazard model. Estimates of hazard ratios for herd milk yield indicate that cows in the low milk yield

category were 0.54 times as likely to become pregnant as were cows from herds in the high category (Table 3). Thus, cows from herds with a higher milk yield category have more chance of getting pregnant.

Discussion

The overall median days open obtained in this study was 154 days. This can produce a calving interval of less than 400 days. However, it is not comparable to a calving interval of 12-13 months, which is suggested as optimal for dairy herds [25]. The median days open found in this study is higher than 109.8 days in Costa Rica [26] and 131 days in Thailand [27], Vietnam, China, and Myanmar (110–118 days) and is comparable with countries such as Bangladesh, Pakistan, Sri Lanka, and Indonesia (147–255 days) [28]. The variation in days open in dairy cattle is mainly determined by factors operating at either the individual cow or lactation level. In addition, the differences could have been by the failure of dairy farmer to detect heat signs after calving. Thus, the calving to insemination interval was prolonged and eventually influencing the days open.

The median days open of dairy cows inseminated in the different seasons were found to be 142, 154, 151, and 169 days for cows inseminated in autumn, winter, spring, and summer, respectively. The days open were significantly differing ($p<0.05$) among the insemination season of cows where the shorter days open was found in autumn inseminated cows than cows inseminated in other seasons, which is in agreement with the finding of [29]. This might be attributed to the positive influence of the autumn season on the reproductive performance of cows via optimal environments and the availability of fresh green pastures through the early lactation period [29] and an improved health condition could explain this finding. Although cows are not typically classified as

seasonal breeders, changes in photoperiodic stimulation [30] associated with specific times of the year provide a potential explanation for such an effect of season on the reproductive measures [31].

The significant effects ($p<0.05$) of the season of calving on days open reveal the seasonal influences on reproduction could include not only climatological changes associated with each season but also nutritional and management factors that are altered on a seasonal basis [32,33]. Also, poor quality feeds obtained during the dry periods resulted in longer days open for cows that calved during those periods because animals take a longer time to recover after calving.

The calving to insemination interval was proved to be an important index for evaluating postpartum conception in dairy cows. In this study, the calving to insemination interval factor has significantly ($p<0.05$) affected days open of dairy cows. Accordingly, the dairy cow with smaller calving to insemination interval (<80 days) is more likely (HR= 2.5) to become pregnant than cows with large calving to insemination interval (>= 80 days). The results from other studies suggest a curvilinear response with conception rates lower amongst cows where calving to service interval is very long [22,34,35], which agrees with our finding. This may be due to selection biases with other factors amongst cows whose first service was delayed causing lower conception rates. Although delaying the first service may increase conception rates, this strategy may improve primary measures of reproductive performance. Contrary to our results, the conception rate was lower in cows with short calving to insemination interval in a Spanish study [36]. These discrepancies might be due to the different categories of the interval from calving to insemination used in each study.

Our findings suggest that herd milk yield levels resulted in considerable effect (p<0.05) on days open (Table 3). The estimated hazard ratios indicate that cows in the low category were less likely (HR=0.54) to be pregnant than were cows from herds in the high category (Table 3). This finding contradicts Eicker et al. [37] who has shown that the effect of milk yield on conception rate is minimal. This might be due to the level of milk yield in that study was considered a continuous variable. In addition, Herman et al. [7] revealed a higher chance of conception for heifers from herds with lower milk yield. In theory, cows with a high milk yield are likely to have more days open because of the negative effect of milk yield on energy balance and reproductive performance. Our result does not fully support this effect because cows in the highest milk yield category showed a higher chance to become pregnant, and the difference among the herd milk yield level was not clear. The lower conception rate in low-producing cows might be caused by proper management decisions on the farm. Milk yield is an important factor in breeding decisions on most dairies [37]. Farmers likely do not show the same interest in breeding low-yielding cows.

Artificially inseminated cows had a higher probability of becoming pregnant than cows exposed to natural service. These results of higher hazard rates for pregnancy in AI exposed cows are consistent with the report of Overton and Sischo [38]. This result is not unforeseen as many dairy farms with bullpens may move cows that experience calving-related trauma and reproductive tract injuries directly into bullpens, or will move them when cows are believed to have fertility problems. Dairy farmers reported that cows are moved from AI into bullpens when the cow is a repeat breeder, extended days in milk (DIM), and poor milk production. This suggests that the dairy producers believe that bull

breeding improves the likelihood that these non-pregnant and potentially lower producing cows will become pregnant, and that the natural service (NS) breeding system lowers their input costs to these less productive cows [38].

The days open of dairy cows in Dessie and Kombolcha town of the study area was not significantly different ($p>0.05$) which is probably because the areas have similar nutritional and health management and productivity of cows [1].

The effect of pre(post)partum disease in this study was inconclusive; although a significant effect ($p>0.05$) of peri(post)partum disease was not observed which is likely due to the small number of cows with reported pre(post)partum disease.

Conclusions

In conclusion, the season of insemination, calving season, breeding system, the calving to insemination interval, and herd milk yield level appears to have a significant effect ($p<0.05$) on days open. As a result, dairy cows calved and inseminated in the autumn and winter season of the year were more likely to be pregnant or decrease the days open, respectively. In addition, cows with shorter calving to insemination interval and artificial insemination exposed tend to have a greater probability to conceive than cows with longer calving to insemination interval and natural service exposed cows. Herd milk yield seemed to affect the days open. Herds with higher milk yield had a higher chance of being pregnant; however, management practices and breeding decisions seem to be more important for the situation analyzed here because the herds with the lowest yield had the lowest chance of getting pregnant.

Supporting information

S1 File. dataset

Acknowledgments

The authors gratefully acknowledge dairy farm owners at Dessie and Kombolcha towns for allowing us to conduct this study and follow-up their animals during the study period.

References

1. Kim IH, Jeong JK. Risk factors limiting first service conception rate in dairy cows and their economic impact. Asian-Australasian J Anim Sci. 2019;32(4):519–26.
2. Pryce JE, Veerkamp RF, Thompson R, Hill WG, Simm G. Genetic aspects of common health disorders and measures of fertility in Holstein Friesian dairy cattle. Anim Sci. 1997;65(3):353–60.
3. Yusuf M, Nakao T, Yoshida C, Long ST, Gautam G, Bimalka Kumari Ranasinghe RMS, et al. Days in milk at first AI in dairy cows; its effect on subsequent reproductive performance and some factors influencing it. J Reprod Dev. 2011;57(5):643–9.
4. Inchaisri C, Jorritsma R, Vos PLAM, van der Weijden GC, Hogeveen H. Economic consequences of reproductive performance in dairy cattle. Theriogenology. 2010;74(5):835–46.
5. Grimard B, Freret S, Chevallier A, Pinto A, Ponsart C, Humblot P. Genetic and environmental factors influencing first service conception rate and late embryonic/foetal mortality in low fertility dairy herds. Anim Reprod Sci.

2006;91(1–2):31–44.

6. Tillard E, Humblot P, Faye B, Lecomte P, Dohoo I, Bocquier F. Postcalving factors affecting conception risk in Holstein dairy cows in tropical and sub-tropical conditions. Theriogenology. 2008;69(4):443–57.

7. Harman JL, Casella G, Gröhn YT. The application of event-time regression techniques to the study of dairy cow interval-to-conception. Prev Vet Med. 1996;26(3–4):263–74.

8. Cabrera VE. Economics of fertility in high-yielding dairy cows on confined TMR systems. Animal. 2014;8(SUPPL. 1):211–21.

9. González-Recio O, Pérez-Cabal MA, Alenda R. Economic value of female fertility and its relationship with profit in Spanish Dairy Cattle. J Dairy Sci. 2004;87(9):3053–61.

10. Louca A, Legates JE. Production Losses in Dairy Cattle Due to Days Open. J Dairy Sci. 1968;51(4):573–83.

11. Allison PD. Survival Analysis Using SAS: A practical Guide, Second Edition. Vol. 36, Technometrics. 2010. 125–201 p.

12. Allore HG, Warnick LD, Hertl J, Gröhn YT. Censoring in survival analysis: A simulation study of the effect of milk yield on conception. Prev Vet Med. 2001;49(3–4):223–34.

13. Berry DP, Lee JM, Macdonald KA, Stafford K, Matthews L, Roche JR. Associations Among Body Condition Score, Body Weight, Somatic Cell Count, and Clinical Mastitis in Seasonally Calving Dairy Cattle. J Dairy Sci. 90:637–48.

14. Suriyasathaporn W, Nielen M, Dieleman SJ, Brand A, Noordhuizen-Stassen EN, Schukken YH. A Cox proportional-hazards model with time-dependent covariates

to evaluate the relationship between body-condition score and the risks of first insemination and pregnancy in a high-producing dairy herd. Prev Vet Med. 1998;37(1–4):159–72.

15. Clark TG, Bradburn MJ, Love SB, Altman DG. Survival Analysis Part I: Basic concepts and first analyses. Br J Cancer. 2003;89(2):232–8.

16. Kim KD, Ki KS, Kang HG, Kim IH. Risk factors and the economic impact of ovarian cysts on reproductive performance of dairy cows in Korea. J Reprod Dev. 2005;51(4):491–8.

17. R Core Team. R: The R Project for Statistical Computing [Internet]. 2019 [cited 2021 Feb 19]. Available from: https://www.r-project.org/

18. Lumley T, S- R, Elizabeth A, Cynthia C, Therneau MTM. Package ‘ survival .’ 2021;

19. Package T. Package ‘ GGally .’ 2021;

20. Cox DR. Regression Models and Life-Tables. J R Stat Soc Ser B. 1972;34(2):187–202.

21. Martin AD, Kielland C, Nelson ST, Østerås O. The effects of building design on hazard of first service in Norwegian dairy cows. J Dairy Sci. 2015;98(12):8655–63.

22. Vargas B, Van Der Lende T, Baaijen M, Van Arendonk JAM. Event-Time Analysis of Reproductive Traits of Dairy Heifers. J Dairy Sci. 1998;81(11):2881–9.

23. Loeffler SH, De Vries MJ, Schukken YH. The effects of time of disease occurrence, milk yield, and body condition on fertility of dairy cows. J Dairy Sci. 1999;82(12):2589–604.

24. Lombard JE, Garry FB, Tomlinson SM, Garber LP. Impacts of dystocia on health and survival of dairy calves. J Dairy Sci. 2007;90(4):1751–60.

25. Sheldon IM, Lewis GS, LeBlanc S, Gilbert RO. Defining postpartum uterine disease in cattle. Theriogenology. 2006;65(8):1516–30.

26. Radostitis, O. and Blood D. A textbook of the diseases of cattle, horses, sheep, pigs and goats. 10th editi. Otto Radostits Clive Gay Kenneth Hinchcliff Peter, editor. 2001. 2180 p.

27. Vargas B, Van Der Lende T, Baaijen M, Van Arendonk JAM. Event-Time Analysis of Reproductive Traits of Dairy Heifers. J Dairy Sci. 1998;81(11):2881–9.

28. Punyapornwithaya V. Reproductive efficiency of dairy cows in the northern part of Thailand Original article. 2020;(April 2004).

29. García SC, Holmes CW. Lactation curves of autumn- and spring-calved cows in pasture-based dairy systems. Livest Prod Sci. 2001;68(2–3):189–203.

30. Hammoud M, El-Khateeb S, Oudah E. Effect of sire, age at first calving, season and year of calving and parity on reproductive performance of Friesian cows under semiarid conditions in Egypt. Arch Zootech. 2010;13(1):60–82.

31. Dahl GE, Buchanan BA, Tucker HA. Photoperiodic effects on dairy cattle: A review. J Dairy Sci. 2000;83(4):885–93.

32. Santos JEP, Rutigliano HM, Filho MFS. Risk factors for resumption of postpartum estrous cycles and embryonic survival in lactating dairy cows. Anim Reprod Sci. 2009;110(3–4):207–21.

33. Zakari AY, Molokwu ECI, Osori DIK. Effects of rectal and ambient temperatures and humidity on conception rates. Theriogenology. 1981;16(3):331–6.

34. Francos G, Mayer E. Observations on some environmental factors connected with fertility in heat stressed cows. Theriogenology. 1983;19(5):625–34.

35. Thompson JA, Magee DD, Tomaszewski MA, Wilks DL, Fourdraine RH. Management of summer infertility in texas holstein dairy cattle. Theriogenology. 1996;46(3):547–58.

36. Loeffler SH, De Vries MJ, Schukken YH, De Zeeuw AC, Dijkhuizen AA, De Graaf FM, et al. Use of AI technician scores for body condition, uterine tone and uterine discharge in a model with disease and milk production parameters to predict pregnancy risk at first AI in holstein dairy cows. Theriogenology. 1999;51(7):1267–84.

37. Portas V, Láctea P, Retrospectivo E, El EN, España NDE. How to cite Complete issue More information about this article RISK FACTORS FOR LOW PREGNANCY RATE IN DAIRY CATTLE : A RETROSPECTIVE STUDY IN THE NORTH WEST OF SPAIN. 2004;

38. Eicker SW, Gröhn YT, Hertl JA. The Association between Cumulative Milk Yield, Days Open, and Days to First Breeding in New York Holstein Cows. J Dairy Sci. 1996;79(2):235–41.

39. Overton MW, Sischo WM. Comparison of reproductive performance by artificial insemination versus natural service sires in California dairies. Theriogenology. 2005;64(3):603–13.

 Click here to access/download;Figure;Fig 1.tif

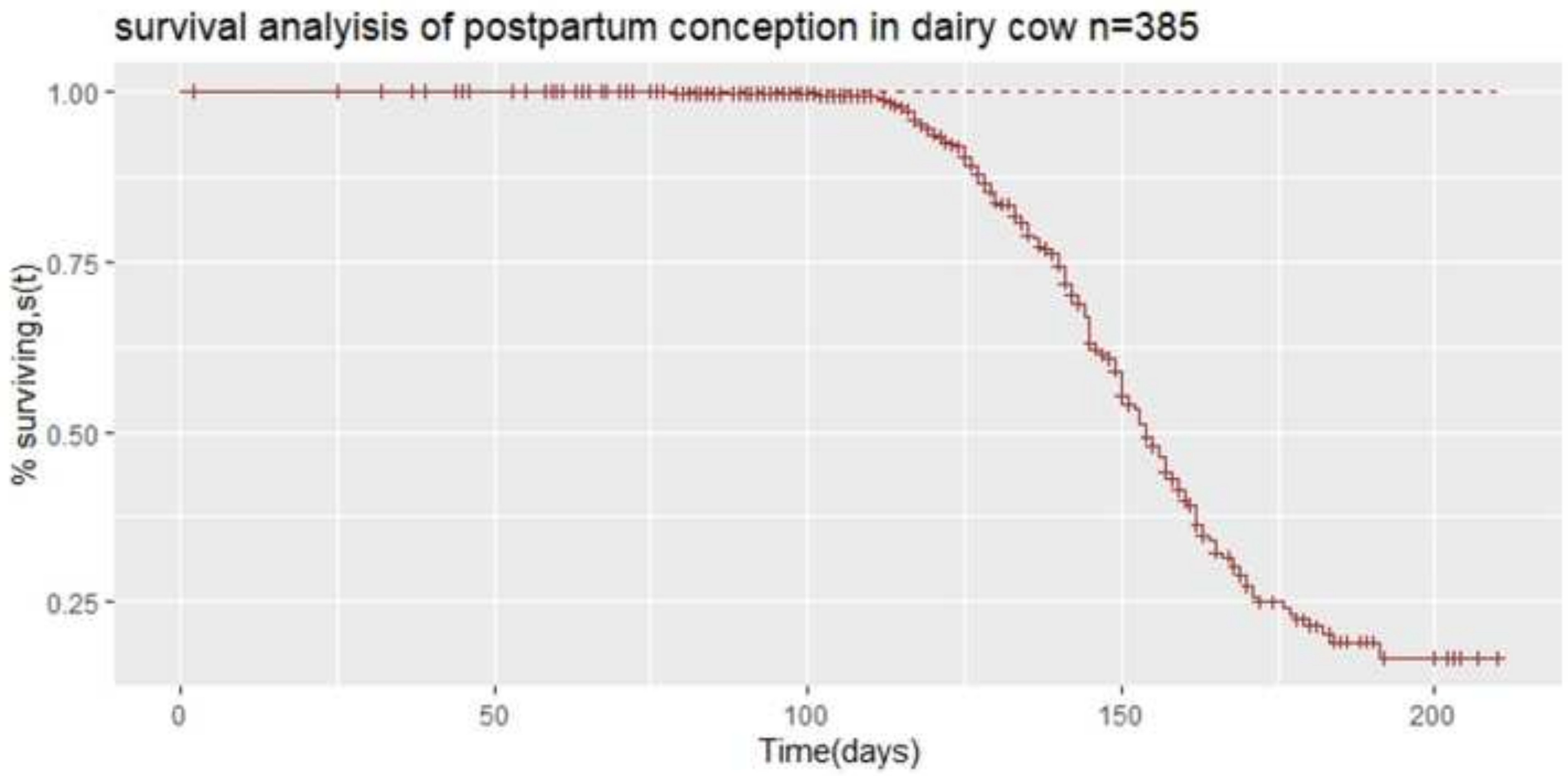

Figure

Click here to access/download;Figure;Fig 2.tif

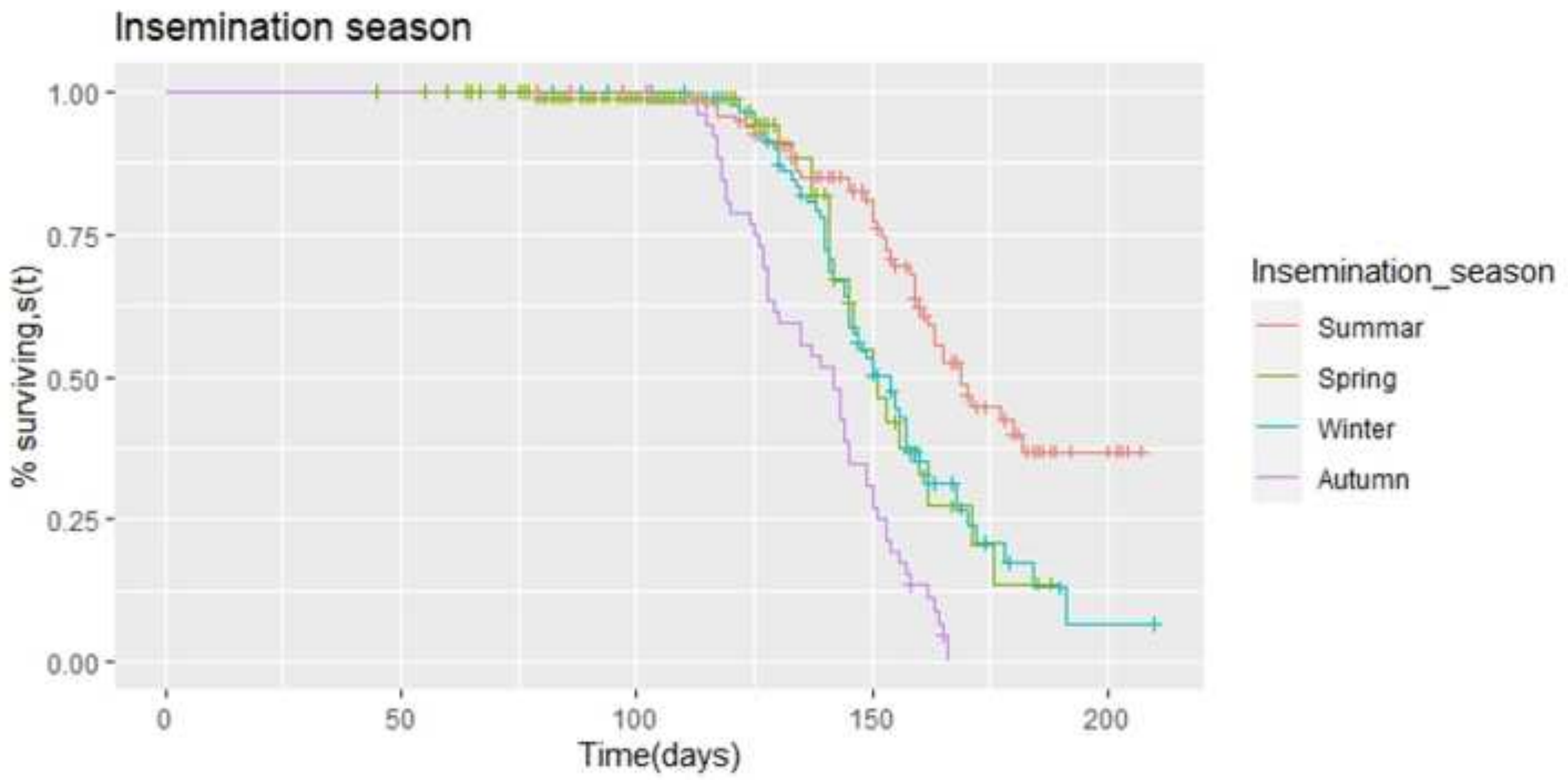

Click here to access/download;Figure;Fig 3.tif

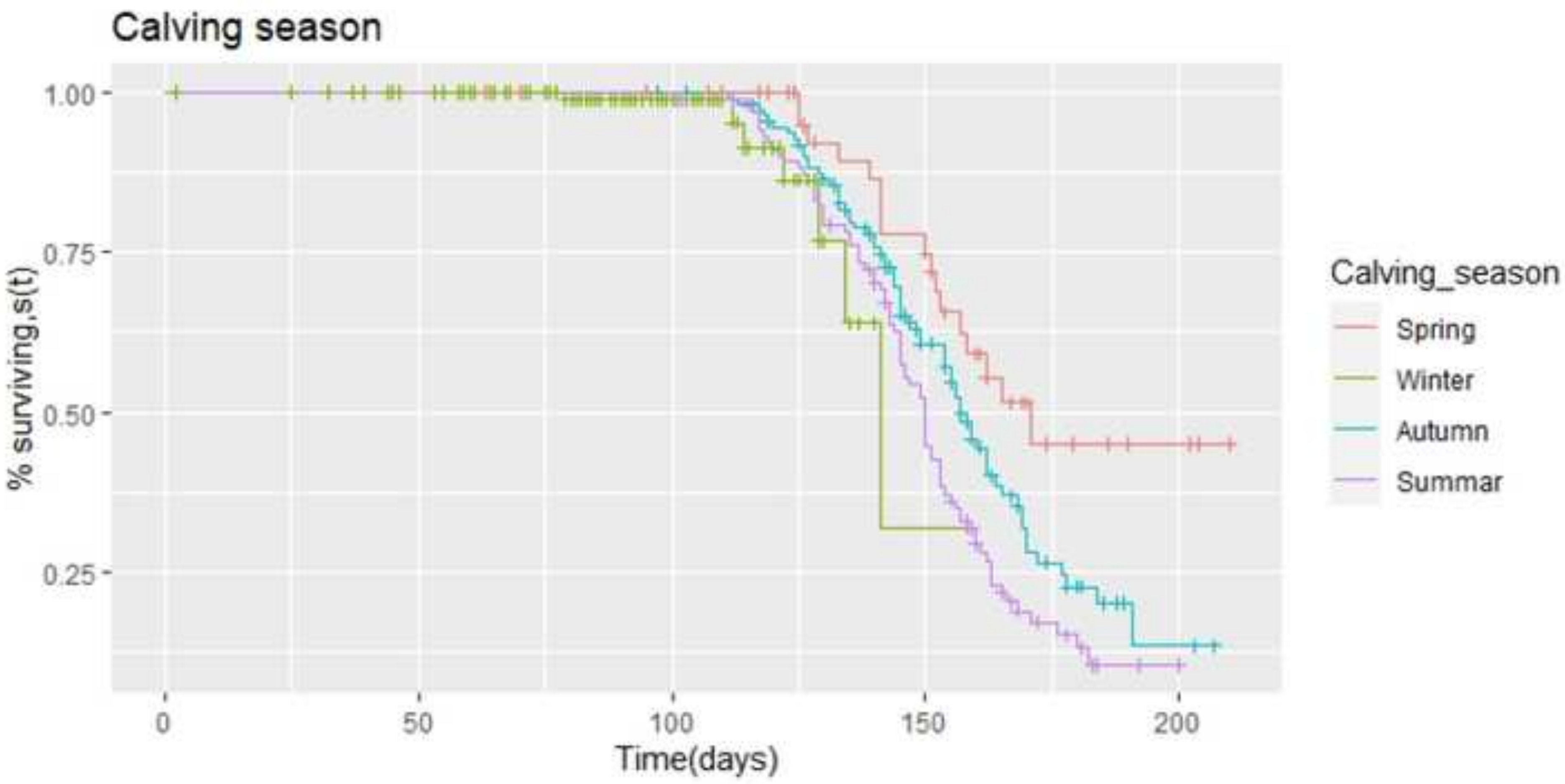

 Click here to access/download;Figure;Fig 4.tif

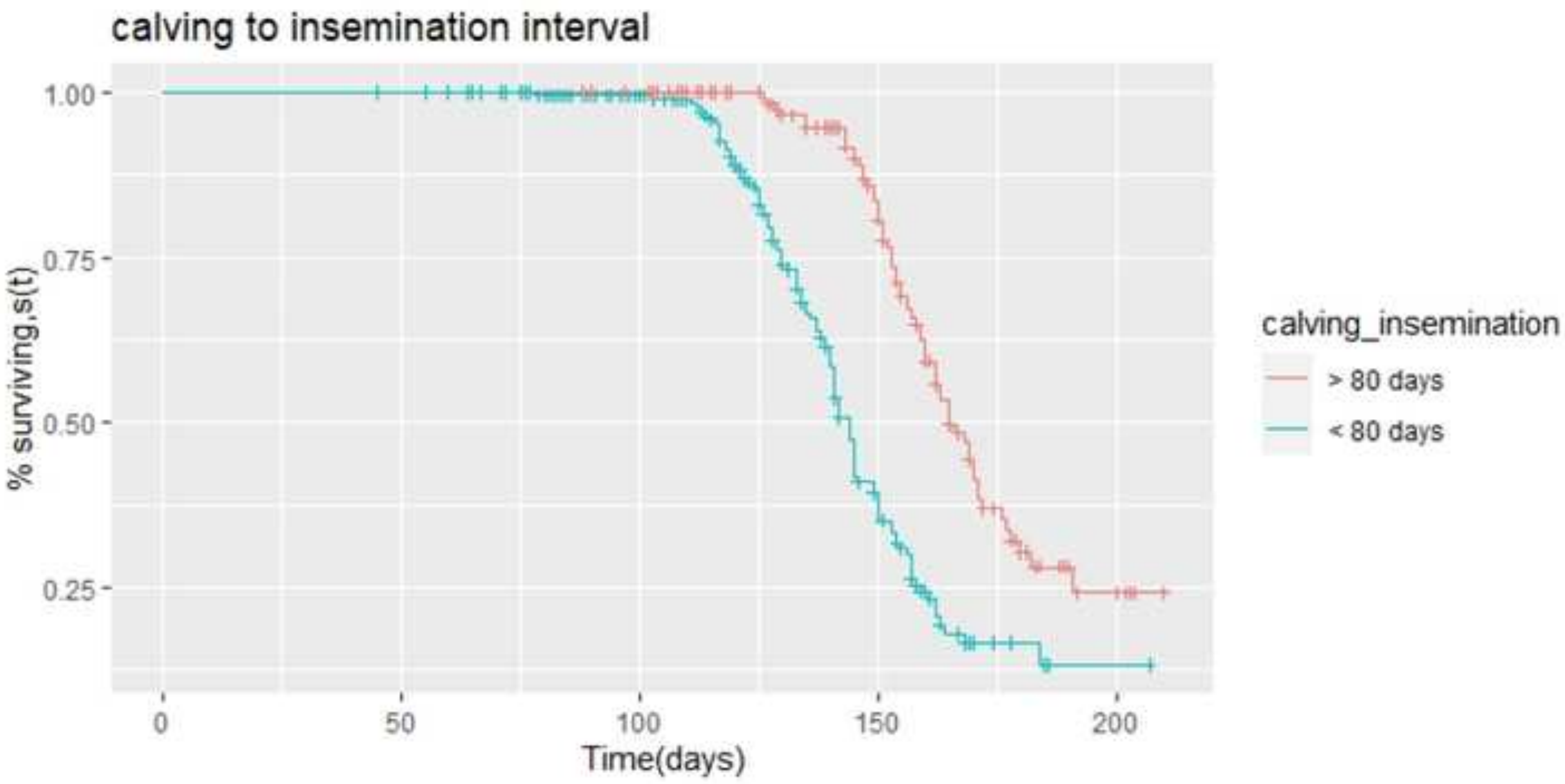

Figure

Click here to access/download;Figure;Fig 5.tif

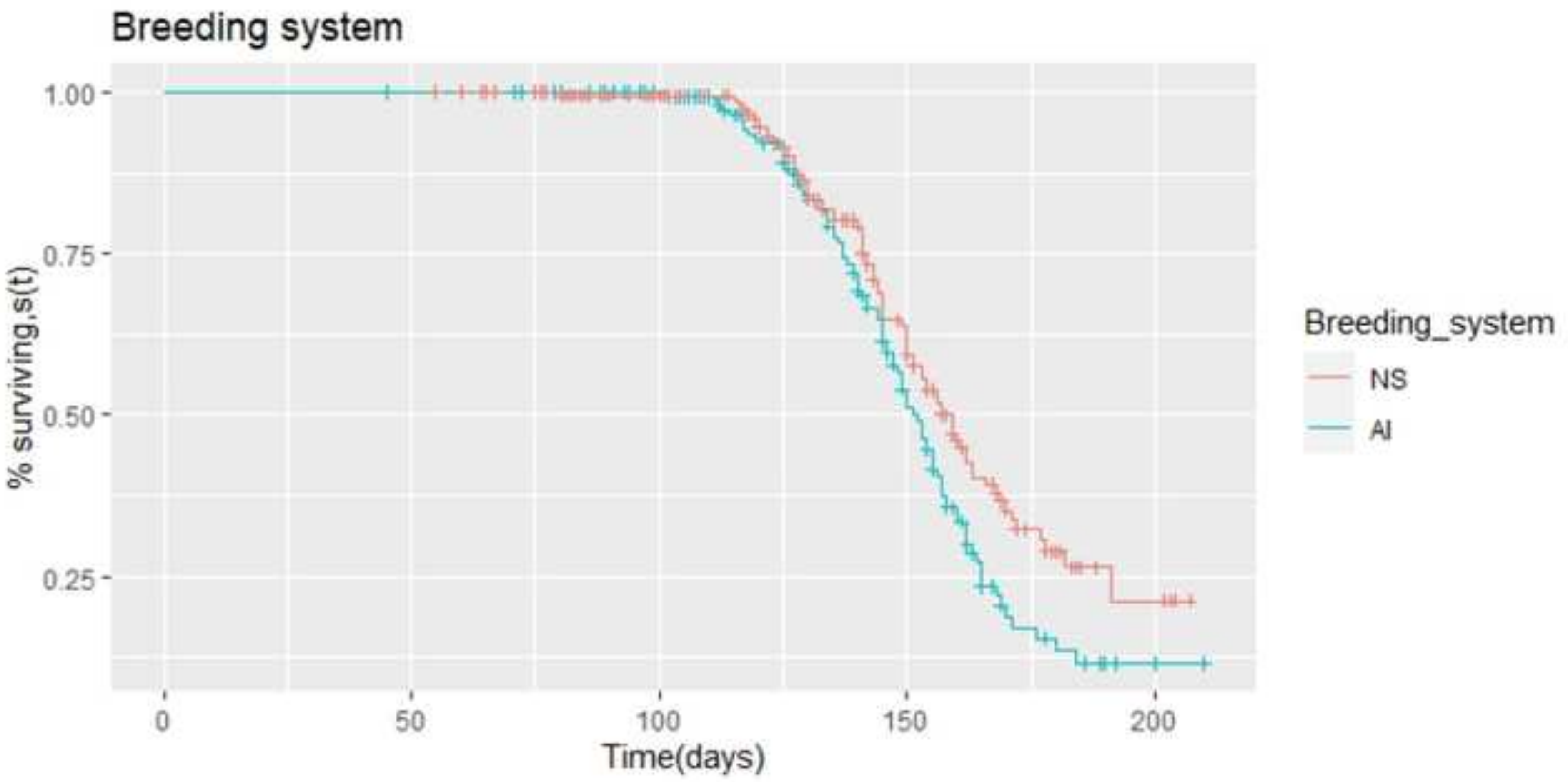

Click here to access/download
Supporting Information
S1_File.xls
